# Contrast Microsphere Destruction by a Continuous Flow Ventricular Assist Device: An In Vitro Evaluation Using a Mock Circulation Loop

**DOI:** 10.1155/2017/4907898

**Published:** 2017-08-13

**Authors:** David G. Platts, Nicole Bartnikowski, Shaun D. Gregory, Gregory M. Scalia, John F. Fraser

**Affiliations:** ^1^Department of Echocardiography, The Prince Charles Hospital, Brisbane, QLD, Australia; ^2^School of Medicine, University of Queensland, Brisbane, QLD, Australia; ^3^Innovative Cardiovascular Engineering and Technology Laboratory, Critical Care Research Group, The Prince Charles Hospital, Brisbane, QLD, Australia; ^4^School of Chemistry, Physics and Mechanical Engineering, Queensland University of Technology, Brisbane, QLD, Australia; ^5^School of Engineering, Griffith University, Brisbane, QLD, Australia; ^6^Heart Care Partners, Wesley Hospital, Brisbane, QLD, Australia; ^7^Adult Intensive Care Service, The Prince Charles Hospital, Brisbane, QLD, Australia

## Abstract

**Objectives:**

Transthoracic echocardiography (TTE) is fundamental in managing patients supported with ventricular assist devices (VAD). However imaging can be difficult in these patients. Contrast improves image quality but they are hydrodynamically fragile agents. The aim was to assess contrast concentration following passage through a VAD utilising a mock circulation loop (MCL).

**Methods:**

Heartware continuous flow (CF) VAD was incorporated into a MCL. Definity® contrast was infused into the MCL with imaging before and after CF-VAD. 5 mm^2^ regions of interest were used to obtain signal intensity (decibels), as a surrogate of contrast concentration.

**Results:**

Four pump speeds revealed significant reduction in contrast signal intensity after CF-VAD compared to before CF-VAD (all *p* < 0.0001). Combined pre- and postpump data at all speeds showed a 22.2% absolute reduction in contrast signal intensity across the CF-VAD (14.8 ± 0.8 dB prepump versus 11.6 ± 1.4 dB postpump; *p* < 0.0001). Mean signal intensity reduction at each speed showed an inverse relationship between speed and relative reduction in signal intensity.

**Conclusion:**

Contrast microsphere transit through a CF-VAD within a MCL resulted in significant reduction in signal intensity, consistent with destruction within the pump. This was evident at all CF-VAD pump speeds but relative signal drop was inversely proportional to pump speed.

## 1. Introduction

Continuous flow ventricular assist devices (CF-VADs) are utilised to help manage selected patients with severe heart failure [1–3]. With improvements in device design and patient selection, CF-VADs are playing an increasing role in the management of heart failure and the implant rates have risen significantly over the last few years [4, 5]. They are typically used as either a bridge to cardiac transplantation or with increasing frequency, as destination therapy [6, 7]. Echocardiography has a fundamental role in the management of patients supported with a CF-LVAD. This starts with assisting in patient selection and sequentially progresses through assisting with implantation, optimising pump parameters, patient monitoring, detection of complications, and finally assessment for cardiac recovery [8–12].

There are numerous echocardiographic modalities available to image patients with a CF-VAD, with selection dependent upon multiple factors, including indication, image quality, and patient location. Transthoracic echocardiography (TTE) is usually the initial form of scanning performed in the assessment of these patients as it is a safe, bedside imaging modality that provides structural and functional information if the image quality is adequate [13–15]. However, a significant number of TTE studies, especially in the critical care complex, are nondiagnostic due to suboptimal image quality. Common reasons for this include limitation in accessing or optimising conventional TTE views due to inability to alter patient positioning, presence of surgical drains or dressings, and suboptimal lighting conditions [16, 17]. Transesophageal echocardiography (TEE) can overcome many of these limitations. Patients supported with a VAD may however have a contraindication to invasive esophageal intubation such as a coagulopathy or respiratory compromise, especially in the early stages of their support. The addition of an echocardiographic contrast agent coupled with contrast enhanced imaging modalities can reduce the number of nondiagnostic TTE studies [18–22] and potentially obviate the need for invasive TEE. However, as these contrast agents are engineered microspheres, they are hydrodynamically labile and prone to destruction when exposed to turbulent flow and high shear forces [23, 24]. Within a CF-VAD system, there are several factors which may promote increased bubble destruction, including direct physical trauma and major intradevice pressure changes. The aim of this study was to determine the impact that transit through a CF-VAD device had on contrast microsphere concentration, utilising a validated mock circulation loop (MCL).

## 2. Materials and Methods

This research utilised a fluid-filled MCL in which a CF-VAD was incorporated and operated at multiple, clinically relevant pump speeds. An echocardiographic contrast agent was infused into the circuit and contrast specific imaging was performed before CF-VAD and after CF-VAD. Contrast signal intensities, acting as a surrogate of contrast concentration, were then analysed to determine contrast degradation across the pump.

### 2.1. Mock Circulation Loop

**Figure 1:**
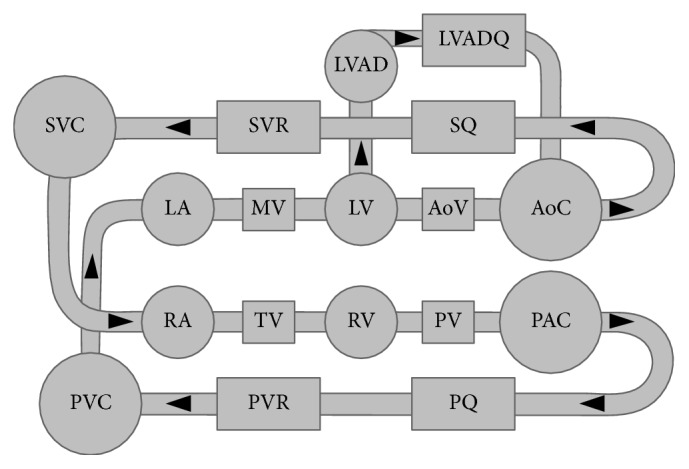
Schematic of the mock circulation loop used for this study. LA: left atrium; MV: mitral valve; LV: left ventricle; AoV: aortic valve; AoC: systemic arterial compliance; SQ: systemic flow meter; SVR: systemic venous resistance; SVC: systemic venous compliance; RA: right atrium; TV: tricuspid valve; RV: right ventricle; PV: pulmonary valve; PAC: pulmonary arterial compliance; PQ: pulmonary flow meter; PVR: pulmonary venous resistance; PVC: pulmonary venous compliance; LVAD: left ventricular assist device.

A detailed description of the MCL used in this research can be found elsewhere [25, 26]. Briefly, this MCL consists of an in vitro simulation of the cardiac and circulatory systems. Clear polyvinyl chloride (PVC) pipes orientated vertically represent the right and left atria and right and left ventricles. Horizontally placed and connected PVC pipes represent the arterial and venous circulations, with systemic and pulmonary vascular resistances maintained at 1300 and 100 dyne·s·cm^−5^, respectively throughout the experiment via variable resistance valves (VMP025.03X.71, AKO, Alb. Klein Ohio LLC, USA). There are also four mechanical flap valves that simulate the four native cardiac valves. Systemic and pulmonary arterial and venous compliance were simulated with lumped, air-filled Windkessel chambers. The MCL is filled with a fluid consisting of a water/glycerol mixture (60/40% by mass). At normal operating room temperature of 22°C, this has a similar viscosity (3.5 mPa·s) and density (1100 kg·m^−3^) to that of blood at 37°C. Incorporated within the systemic component of the MCL was a Heartware® CF-VAD (Heartware, Framingham, MA, USA) attached to the left ventricle (inflow) and ascending aorta (outflow). Inflow and outflow cannulae were represented by 500 mm length, 12.5 mm inner diameter, and 1.6 mm wall thickness Tygon tubing to allow the flow to fully develop by the imaging point.  Figure 1 is a schematic diagram of the MCL.

The Heartware CF-VAD was controlled via the standard Heartware monitor, which enabled pump speed adjustment. The CF-VAD was operated at four different clinically relevant pump speeds: 2400, 2800, 3200, and 3600 revolutions per minute (RPM). Flow through the CF-VAD was measured using a clamp-on ultrasonic flow meter (TS410-10PXL, Transonic Systems, Ithaca, NY, USA). Pressures in the MCL were measured using silicone-based pressure transducers (PX181B-015C5V, Omega Engineering, Stamford, CT, USA). Pressure and flow data was recorded at 100 Hz using a dSPACE acquisition system (DS1103, dSPACE, Wixom, MI, USA). A total heart failure condition was simulated with no ventricular contractility to produce a continuous flow through the MCL and CF-VAD.

### 2.2. Echocardiographic Contrast Agent

One ampoule of the perflutren microsphere, Definity (Lantheus Medical imaging, Billerica, MA, USA) was activated and diluted to 50 millilitres (mL) with normal saline. Following activation, this echocardiographic contrast agent has a mean bubble diameter of 1.1–3.3 microns and undiluted; each 1 mL of activated contrast has a maximum of 1.2 × 10^10^ Definity microspheres [27]. The contrast agent was infused via a minimum volume extension line at 250 mL/hour using an Alaris GH Plus infusion pump (CareFusion, San Diego, CA, USA). The infusion point was proximal to the CF-VAD, via an access side port close to the left ventricle of the MCL. The contrast solution was regularly agitated to ensure even delivery of contrast microspheres.

### 2.3. Contrast Imaging Acquisition

A Philips iE33 ultrasound scanner and an S5-1 MHz ultrasound transducer were used to acquire the echocardiographic images. A custom made silicone ultrasound “stand-off,” with a 5 mm spacer between the outer tube surface and flat probe seat, was applied at both of the scanning points on the tubing of the MCL. Imaging with a contrast specific modality was commenced when there was a steady state of contrast within the circuit. The contrast imaging utilised was a low mechanical index, multipulse, power inversion technique (power modulation) using an imaging frequency of 2 MHz, mechanical index of 0.1, gain at 50%, fixed focal zone, depth of 50 mm, compression of 50, and frame rate of 39 Hz. Persistence was always set to “off.” These settings remained constant for all images acquired. This was to remove any system setting changes that could account for amplitude changes in signal intensity in the time intensity displays.

Echocardiographic imaging was performed both proximal (“prepump”) and distal (“postpump”) to the CF-VAD within the MCL, at all pump speeds (2400, 2800, 3200, and 3600 RPM) and at room temperature. Following any adjustment in CF-VAD pump speed, image acquisition was paused to enable a new steady state in contrast concentration within the circuit. The prepump images were acquired immediately proximal (approximately 20 mm) to the CF-VAD inflow cannula. The postpump images were acquired 300 mm distal to the CF-VAD outflow cannula. Two second ultrasound clips were acquired for all data points, resulting in 78 ultrasound frames for each analysis. The MCL was then returned to baseline state and all data points at all pump speeds were then repeated with a second infusion of contrast at the same infusion rate and concentration.

### 2.4. Contrast Imaging Analysis

**Figure 2:**
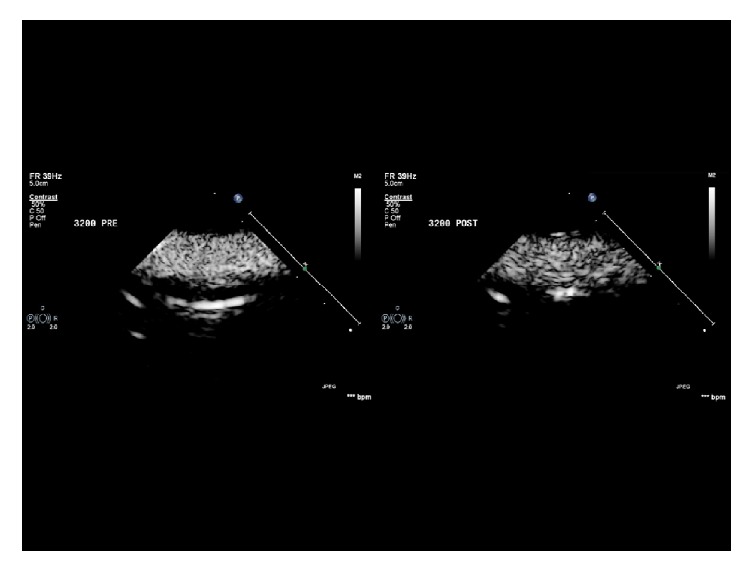
Pre-CF-VAD and post-CF-VAD contrast image within the MCL at a pump speed of 3200 RPM.

**Figure 3:**
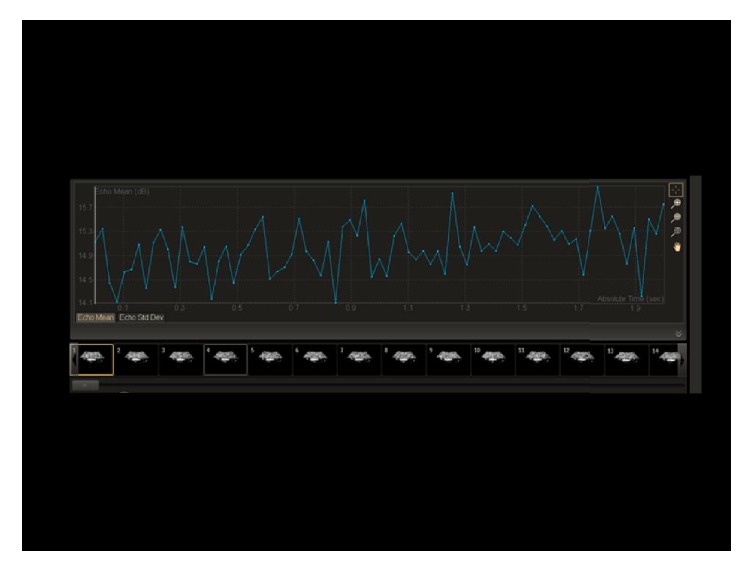
Example of a contrast clip and the resultant contrast signal intensity graph over time.

The contrast images were acquired in raw data format and transferred to a workstation for analysis using QLAB (Philips Medical Systems, Amsterdam, Netherlands). A prespecified 5 mm^2^ region of interest (ROI) was positioned within the centre of the contrast image. Within this ROI, the mean signal intensity in decibels (dB) was measured throughout the 2-second clip. As the signal intensity is a surrogate of contrast microsphere concentration [28, 29], any destruction of contrast within the CF-VAD could be calculated by comparing pre and postpump values.  Figure 2 demonstrates a pre-CF-VAD and a post-CF-VAD contrast image within the MCL.  Figure 3 represents an example of a contrast signal intensity graph over time.

### 2.5. Statistical Methodology

The contrast signal intensity was expressed as mean ± 1 standard deviation (SD) for each of the data points. Student's *t*-test was used to compare the pre- versus postpump mean signal intensity. A* p* value of <0.05 was defined as significant. Analysis was performed using GraphPad Prism version 7.0 (GraphPad Software, La Jolla, CA, USA).

## 3. Results

**Figure 4:**
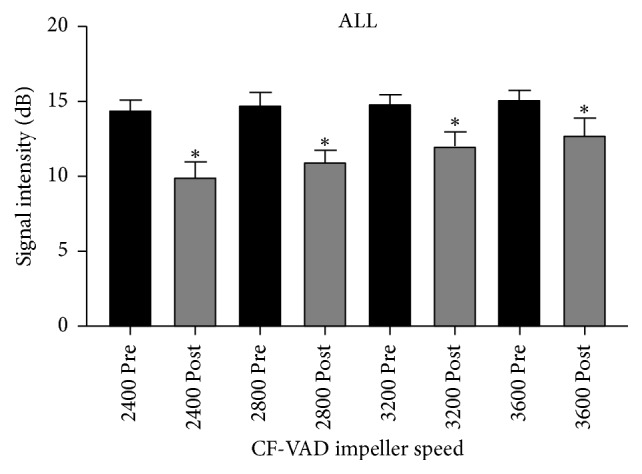
Mean signal intensity prepump and postpump ± 1 SD at each impeller speed (combined data) *∗* represents *p* < 0.0001.

**Figure 5:**
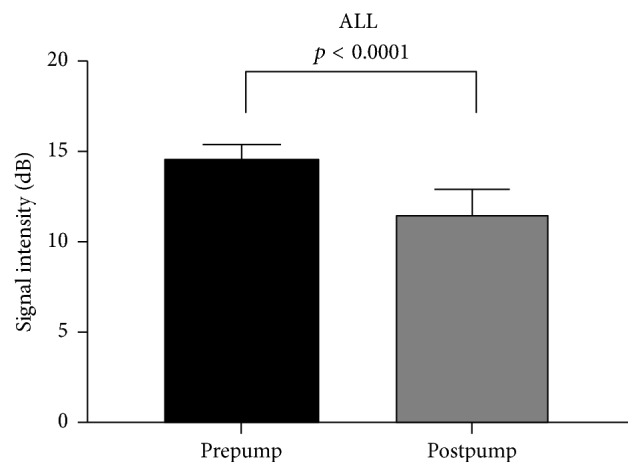
Combined mean signal intensity ± 1 SD for prepump and postpump imaging.

It was technically feasible to acquire contrast specific echocardiographic images at both the pre- and post-CF-VAD at all pump speeds. At all four speeds, there was a significant reduction in contrast signal intensity post-CF-VAD compared to pre-CF-VAD (all *p* < 0.0001). The mean signal intensities ± 1 standard deviation at all four pump speeds pre- and postpump are displayed in Table 1 (run 1), Table 2 (run 2), and Table 3 (combined data).  Figure 4 represents the combined pre- and postpump data at each pump speed. Analysis of combined pre- and postpump data at all speeds (Figure 5) showed that there was a 22.2% absolute reduction in contrast signal intensity across the CF-VAD (14.8 ± 0.8 dB prepump versus 11.6 ± 1.4 dB postpump; *p* < 0.0001).

**Figure 6:**
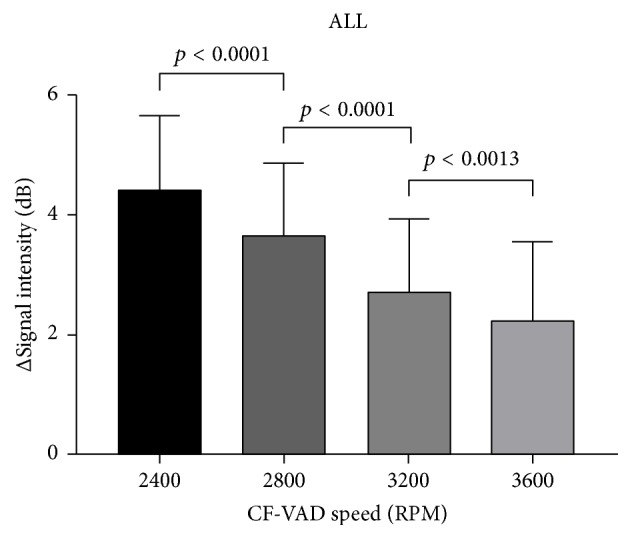
Signal reduction data ± 1 SD at each pump speed (combined data).

CF-VAD flow rates and pressures for pump speeds of 2400, 2800, 3200, and 3600 RPM are shown in Table 4. As expected, increases in pump speed resulted in increased CF-VAD flow rate and postpump pressures whilst prepump pressures decreased. Evaluation of the mean signal intensity reduction across the pump at each pump speed revealed an inverse relationship between speed and relative reduction in signal intensity. This was evident in both run 1 and run 2. The higher the pump speed, the less the drop in contrast signal across the pump. However, this relative drop was statistically significant between each of the comparator speeds.  Figure 6 represents the combined signal reduction data for both pump runs at each pump speed.

## 4. Discussion

There are three key aspects relating to contrast destruction within a CF-VAD that this study has highlighted. Firstly, there was a significant reduction in contrast signal intensity post CF-VAD compared to pre CF-VAD, consistent with contrast destruction within the CF-VAD. Secondly, this significant reduction in contrast signal intensity was evident at all measured CF-VAD pump speeds. Thirdly, with increasing CF-VAD pump speed, there was a relative decrease in the absolute drop in signal intensity across the pump.

There was a significant reduction in contrast signal intensity across the CF-VAD. This was evident at all four pump speeds. Overall there was nearly a 25% reduction in signal intensity following passage of the contrast microspheres through the CF-VAD. With signal intensity obtained via this scanning environment being correlated with contrast concentration, the alteration in signal intensity was acting as a surrogate for contrast destruction during passage through the pump. There are likely to be several reasons to account for this contrast destruction by the CF-VAD. First and foremost, any fluid within a CF-VAD is exposed to significant hydrodynamic forces that are not encountered in the usual physiologic environment. The velocity of fluid flow is significantly greater than usually encountered. The pathway of the fluid is relatively convoluted into, within, and out of the CF-VAD. The fluid is in direct contact with nonbiologic, metallic surfaces and microsphere-impeller contact time may influence bubble destruction levels. There is also a pressure differential within the pump as well as ambient temperature changes. All of these factors increase trauma to the microspheres, resulting in increased destruction.

Evaluation of the individual pump speeds also demonstrated significant reductions in postpump signal intensities at all pump speeds. Of note, however, was the unexpected finding that with increasing pump speeds, the relative difference between prepump and postpump signal intensity decreased. The absolute differences (reduction) however, between pre and postpump values, were significant at each of the three comparison points (2400 versus 2800, 2800 versus 3200, and 3200 versus 3600 RPM). Our model whilst using ROI evaluation of signal intensity did not use a flash destruction replenishment technique as absolute velocity of flow was not the focus of this research and was already known via transducers within the circuit. The key parameter was absolute signal intensity pre- and postpump, which acted as a surrogate of microsphere concentration. It is unlikely that this parameter was significantly altered by flow. Prepump signal intensity values at all pump speeds (and hence flow rates) were relatively constant, reflecting the homogeneous concentration of microsphere contrast after it entered the circuit but before transit through the pump. The flow rate prepump is the same as that postpump in this model. As the prepump signal intensities for all flow rates were relatively constant and the postpump signal intensities varied depending upon pump speed, our inference is that intrapump variables influenced this variation, rather than it being just a simple function of flow. This argument can also be used to exclude simple contrast microsphere recirculation to account for higher postpump signal intensities (the data was collected sequentially starting with the lowest pump speed). As part of insulin-skeletal muscle metabolic research, Ross et al. used an in vitro microdialysis circuit and quantified contrast echocardiography at varied flow rates and similarly found that signal intensities were not influenced by changes in flow [30].

We postulate that there could be several reasons for this inverse relationship between pump speed and drop in contrast concentration across the pump. The first possible reason for a higher postpump signal intensity value with higher pump speeds is contrast transit time. With higher speeds, the contrast has a quicker transit time through the CF-VAD. Assuming that exposure to adverse forces within the CF-VAD results in microsphere destruction, it may be that a shorter exposure time to these forces may actually reduce the microsphere destruction. There was a 50% difference in pump speed between the highest and lowest pump settings. Whilst absolute transit times for a particle at respective speeds are not known, this variation is likely to have a significant effect on absolute transit times. However, countering this argument is at higher pump speeds; one could consider that the hydrodynamic and sheer destructive forces may be greater, resulting in a lower postpump signal intensity value. Another factor that may influence contrast destruction at higher pump speed is possible alterations in the primary, secondary, or tertiary flow paths seen within the Heartware LVAD. Additionally, the impeller gap height within the pump housing may alter sufficiently to impact on microsphere destruction. There is a curvilinear correlation between impeller gap height and pump speed for a Heartware CF-VAD [31]. However, these are only suppositions and our model does not have the ability to proportionate any of these factors on microsphere destruction. Subsequent computational fluid dynamic studies and particle imaging velocimetry will shed further light on the pharmacodynamics properties of contrast microspheres during their transit through a CF-VAD.

To put these findings in context, an understanding of the interplay between the structure and flow profile of the Heartware CF-VAD and the structure and pharmacokinetics of Definity contrast microspheres is required. The Heartware CF-VAD is a centrifugal continuous flow pump [32]. The complete system consists of the pump itself (with an integrated inflow cannula and a 10 mm gortex outflow graft, omitted from this study), driveline, external controller, and a power supply. The Heartware CF-VAD provides blood flow by generating a pressure differential (“pump head”) between the inflow and outflow components of the pump [33].

The actual flow dynamics of blood within the Heartware CF-VAD is complex. Computational fluid dynamics (CFD) analysis has been performed to evaluate blood flow within the Heartware CF-VAD [34]. These show that there are three distinct blood flow paths within the pump. Each of these has a distinct anatomic location. The primary flow path is through the inflow cannula, then into four flow channels within the levitated impeller which then leads to the pump housing or volute, and finally out through the outflow graft. The secondary flow path is designed to promote “washing” of the underside of the impeller and centre post. The flow is from the volute, up under the impeller and through the space between the centre post and impeller. This blood then recirculates into the primary flow path. The tertiary flow path is designed to “cushion” the impeller and wash the hydrodynamic thrust bearing surface of the impeller. The flow is from the impeller flow channels, over the top of the impeller and then recirculates into the primary flow path. After blood has been pumped from the Heartware CF-VAD, blood flow is laminar through a 10 mm gortex graft which is then fashioned as an end to side anastomosis to the aorta [35, 36].

Definity contrast agent is an engineered microsphere that consists of an outer trilipid shell that encapsulates a biologically inert, high molecular weight gas called octafluoropropane. As they have the same intravascular rheology as red blood cells [37], they can act as blood flow and blood volume “tracers.” By enhancing the blood pool signal, they have multiple well accepted clinical indications [38–41]. By utilising the very high back scattering properties of contrast microspheres and considering the high frame rates of analysis possible with contrast specific imaging, it is possible to quantify numerous parameters of the imaged fluid. By placing a predefined region of interest over the flow, a graph of image intensity over time (or time intensity curve, TIC) can be generated. From analysis of these TICs, information can be obtained regarding blood volume and blood flow velocity [42, 43]. However, amplitude based parameters can be affected by variations in system settings, such as gain or mechanical index, as well as variation in the administration of the contrast microspheres [29]. To prevent this from happening in our research, all contrast imaging settings and the contrast administration technique were identical for all data required along with imaging the conduit with the same technique in the same location every time.

There are also numerous factors that can affect quantification of contrast microsphere backscatter. These include the quality of the contrast image, method of contrast administration, and machine settings, such as gain and mechanical index. However, these are not relevant to our controlled laboratory setting. In our model, the time-signal intensity measured in decibels from the predefined ROI fluctuated with each single frame over the 78 frames acquired.  Figure 2 is a demonstration of this phenomenon. This was a result of a combination of two factors. Firstly, following activation of Definity contrast, there is some size variation in microsphere diameter, which directly influences the backscattering properties. Ultrasound backscatter intensity is related to the sixth power of the microsphere radius [42]. Secondly, within the volume of fluid that passed through the ROI over the 2-second period, there would be some frame to frame variation of Definity concentration. Consequently, the mean value of these 78 individual signal intensities was used for each data point.

Contrast microspheres are fragile structures. There are numerous factors that can result in their destruction. Exposure to excessive insonicating ultrasound power is a common cause in the clinical setting. To minimise this, contrast specific imaging modalities use a low mechanical index, typically less than 0.3 [44, 45]. These agents are so fragile that it is recommended that they are administered in line, rather than via a side port, of an intravenous giving set, to minimise traumatic destruction from orthogonal flow [46]. Once destroyed, the microspheres lose their ability to resonate in response to contrast specific imaging and the technique loses its diagnostic applicability. Exposure to other destructive forces such as turbulent flow, shear forces, direct trauma, and pressure changes will also result in contrast microsphere degradation. These later adverse conditions are encountered when using contrast imaging in patients managed with mechanical cardiac support devices. Additionally, other thermodynamic properties of the microsphere carrying medium can also influence their destruction. These include the liquid temperature and the partial pressure of dissolved gases within that medium [47, 48]. There is limited data on the safety and efficacy of contrast echocardiography in patients supported with mechanical cardiac support. It has been shown to be feasible in an ovine ECMO model [49] as well as limited description of its use in the clinical setting [50–53].

## 5. Clinical Translation

There are two findings of this research that may be of relevance with translation to the clinical setting. Firstly, whilst there was numerically a statistically significant drop in contrast signal intensity across the CF-VAD at all pump speeds, this may not be a clinically significant phenomenon. The overall reduction in signal intensity was just under 25%. The postpump signal intensity was still relatively high and the reduction rate still within the range that most likely would enable satisfactory contrast enhanced TTE in the clinical setting. Adjustments in the contrast infusion rate or dilution could be performed to compensate for any anticipated degradation in contrast image quality secondary to any contrast destruction within the pump. Secondly, in the clinical setting there is a wide range of operating pump speeds within a CF-VAD (2400 to 3200 RPM for a Heartware CF-VAD). Our results suggest that, despite this variation, higher pump speeds are not likely to adversely impact contrast enhanced TTE image quality. Even at lower pump speeds, there was still a strong postpump contrast backscatter signal, suggesting feasibility of this technique across a range of CF-VAD operating speeds. There is very limited data assessing the impact of VAD flow on contrast echocardiography and it is a source of on-going research [54, 55].

## 6. Limitations

This research was performed in an in vitro environment under very controlled conditions. The methodology was designed such that contrast signal intensity pre- and postpump could be rapidly and easily measured by scanning Tygon tubing connected to the pump inflow and outflow. This is removed from the clinical environment where there exist multiple other factors that may impact on contrast microsphere rheology, as well as different scanning conditions. As such, this research assessed contrast destruction within the CF-VAD alone and does not take into account other factors that may be present in the clinical setting. Additionally, this model assumed homogenous contrast concentration within the scanned conduit. Whilst not being able to confirm this assumption, the large region of interest placed over the centre of the field of view, in the same positon of every sampling point, may have mitigated any significant variations in contrast concentration during this research. The ideal model for this research would involve simultaneous imaging of pre and postpump locations. However, this approach requires two echo machines and two scanners, which was not logistically possible. To mitigate any significant variation from sequential data collection, one experienced scanner performed the imaging in the same locations with the same method each time, with minimal time delay between the pre and postpump data acquisition. Also, there are numerous CF-VADs currently available and they are broadly classified as either centrifugal flow or axial flow pumps. Each has its own specific flow properties. Our research evaluated a centrifugal pump only. As such our results could not be translated to other CF-VADS, particularly the axial flow systems.

## 7. Conclusion

Passage of echocardiographic contrast microspheres through a CF-VAD within a MCL resulted in a significant reduction in signal intensity, consistent with contrast destruction within the pump. This was evident at all CF-VAD pump speeds but the relative signal drop was inversely proportional to the pump speed. However, despite the numerically significant reduction in signal intensity, all postpump values were still relatively high and, if translated to the clinical setting, likely to facilitate satisfactory contrast enhanced transthoracic echocardiographic imaging.

## Figures and Tables

**Table 1:** Prepump and postpump mean contrast signal intensity (dB) ± 1 SD with mean difference (run 1).

	Prepump SI (dB)	Postpump SI (dB)	Mean difference	
2400 RPM	14.4 ± 0.9	10.3 ± 1.0	4.1 ± 1.3	*p* < 0.0001
2800 RPM	14.3 ± 0.8	11.1 ± 0.7	3.2 ± 1.1	*p* < 0.0001
3200 RPM	14.9 ± 0.6	12.1 ± 0.9	2.7 ± 1.2	*p* < 0.0001
3600 RPM	15.1 ± 0.6	12.8 ± 1.1	2.3 ± 1.3	*p* < 0.0001

**Table 2:** Prepump and postpump mean contrast signal intensity (dB) ± 1 SD with mean difference (run 2).

	Prepump SI (dB)	Postpump SI (dB)	Mean difference	
2400 RPM	14.6 ± 0.7	9.7 ± 0.9	4.8 ± 1.6	*p* < 0.0001
2800 RPM	15.3 ± 0.7	11.1 ± 0.7	4.2 ± 1.1	*p* < 0.0001
3200 RPM	15 ± 0.7	12.2 ± 0.9	2.8 ± 1.2	*p* < 0.0001
3600 RPM	15.3 ± 0.6	13 ± 1.1	2.3 ± 1.3	*p* < 0.0001

**Table 3:** Prepump and postpump mean contrast signal intensity (dB) ± 1 SD with mean difference (combined data).

	Prepump SI (dB)	Postpump SI (dB)	Mean difference	
2400 RPM	14.5 ± 0.7	10.0 ± 1.0	4.4 ± 1.2	*p* < 0.0001
2800 RPM	14.8 ± 0.9	11.1 ± 0.7	3.7 ± 1.2	*p* < 0.0001
3200 RPM	14.9 ± 0.6	12.2 ± 0.9	2.8 ± 1.2	*p* < 0.0001
3600 RPM	15.1 ± 0.6	12.9 ± 1.1	2.3 ± 1.3	*p* < 0.0001

**Table 4:** VAD flow rates and pressures at each pump speed. LVP: left ventricular pressure, MAP: mean aortic pressure.

	VAD flow rate (L/min)	Pre-VAD pressure (LVP), mmHg	Post-VAD pressure (MAP), mmHg
2400 RPM	3.7	17.3	61.5
2800 RPM	4.4	14.7	74.2
3200 RPM	5.2	11.8	88.0
3600 RPM	6.9	6.4	115.2
